# Social cohesion among Syrian and Turkish children, adolescents, and young adults in Turkey: Data from Turkey

**DOI:** 10.1038/s41597-025-05744-9

**Published:** 2025-08-13

**Authors:** Marta Parigi, Nitya Mittal, Sebastian Vollmer

**Affiliations:** 1 Department of Psychological, Pedagogical, Physical Exercise, and Educational Sciences, University of Palermo, Palermo, Italy; 2 Centre for Modern Indian Studies, University of Goettingen, Göttingen, Germany; 3 Department of Economics and Centre for Modern Indian Studies, University of Goettingen, Göttingen, Germany

**Keywords:** Social sciences, Developing world

## Abstract

This data set provides information about basic social-demographic characteristics and social cohesion among young Turkish nationals and Syrian refugees. Information on different dimensions of social cohesion such as belongingness, trust, altruism and social relations, along with socio-demographic information, was collected from a sample of 1305 adolescents and young adults (12–30 years), and 685 children (6–11 years). Data was collected through field interviews between November 2018 – January 2019 in Ankara, Istanbul, and in the bordering cities of Mardin, Gaziantep, Hatay and Şanlıurfa. The sample was selected using a cluster randomised design among the participants of the “Education Program for Syrian Refugees and Host Communities” (BILSY) program conducted by the German Corporation for International Cooperation (GIZ) in Turkey.

## Background and Summary

The civil war in Syria resulted in more than 6.8 million Syrian refugees, nearly half of whom children, seeking asylum primarily in neighbouring Turkey, Jordan, and Lebanon^1,2^. Turkey has become the host of the largest population of Syrian refugees^2^, as these refugees comprise 4.5 percent of Turkey’s total population^3^. The sudden demographic change adversely affected the social cohesion in the country^4–8^. However, studies that focus on perceptions of adults, and those of younger age groups may exhibit variations. Research specifically focused on youth and children finds higher levels of social cohesion among this younger demographic in Jordan^9,10^. This study collected data to measure various dimensions of social cohesion among young Turkish nationals and Syrian refugees living in Turkey^11^. Data was collected in November 2018 – January 2019 in Ankara, Istanbul and the border cities of Mardin, Gaziantep, Hatay and Şanlıurfa for 1305 adolescents and young adults and 685 children.

The Education Program for Syrian Refugees and Host Communities (BILSY) program was implemented from 2016 to 2019 with a purpose of improving access to formal and informal education for both Syrian refugees and Turkish nationals. This study focuses on the informal component of the program which provided the members of the two communities an opportunity to interact in a positive environment, with the specific aim of enhancing social cohesion. Data for this study was collected from participants of the BILSY program, using a cluster randomised design, to examine the effectiveness of this program in improving social cohesion among Syrian refugees and Turkish nationals^11^.

To summarise, this dataset captures the opinion of a demographic group which has not been studied well in literature but constitutes 60 percent of Syrian refugees in Turkey^12^. A unique aspect of this dataset is that information was collected in an informal setting, unlike other settings that focus on classroom interactions to promote social cohesion^13–15^. The dataset provides granular data on various dimensions of social cohesion (discussed below), thus allowing examination of not just a broad measure of social cohesion but also of each individual component that contributes to overall measure of social cohesion. Since there is no universal definition of social cohesion, its measurement varies across studies, making it difficult to draw comparisons. The detailed information presented in this dataset would ease comparison with other studies measuring social cohesion, and can contribute to better understanding of common ways to measure the concept. A limitation of the dataset is that detailed socio-economic-demographic information about the respondents was not collected to keep the cognitive load minimal. This limits the understanding of the characteristics of the sample and the relationship with various measures of social cohesion. Another limitation is that there are not enough observations or details on the characteristics of the events to study the effectiveness of different types of events (explained below) in improving social cohesion independently. The events varied by duration and type of activity. Some activities were more engaging as compared to others, such as playing a team sport vs. a painting class, and understanding how the characteristics of the activity itself can affect social cohesion would have been useful for planning similar programs in future.

## Methods

This study focuses on the non-formal education component of the BILSY program. Volunteers from both Turkish and Syrian backgrounds underwent training facilitated by local and international social workers. These volunteers subsequently planned and organised social, cultural, and sporting activities, such as picnics, reading clubs, photography clubs and football, for children and youths. These activities were called events. Data for this study was collected through a primary survey between November 2018 and January 2019 in Ankara, Istanbul and the border cities of Mardin, Gaziantep, Hatay and Şanlıurfa.

This study was evaluated by the ethics committee at the University of Göttingen, Germany. The data collection was funded by the German Corporation for International Cooperation (GIZ). Data was collected from the participants of the BILSY program conducted by GIZ in Turkey using a cluster randomised design. GIZ develops its program portfolio in close collaboration with local governments and maintains close communication with them during program implementation. Its collaboration with the Turkish Government for the BILSY project was formalised through a Memorandum of Understanding (MoU) with the Republic of Turkey Ministry of Youth and Sport, and MoUs with the provincial governments. These MoUs granted formal permission to conduct all activities, also including the permission to conduct this evaluation, and therefore approval from local ethics committee was not requested.

### Sample selection

For selecting the sample, we employed a cluster randomised design at the event-level. First, for each age group a comprehensive list of all planned events and the number of participants in each event within the sampling area during the data collection period was compiled. Then using the Probability Proportional to Size (PPS) method, events were randomly selected to be included in the survey.

In total, 60 events for adolescents and young adults, and 27 events for children were randomly selected from the comprehensive list. Each of the chosen events was then randomly assigned to either the control or treatment group. Surveys were conducted with the control group prior to the commencement of the event, while the treatment group was surveyed at the conclusion of the event. This approach allowed us to evaluate the immediate impact of participation in BILSY program events on social cohesion. Within each event, we again employed the Probability Proportional to Size (PPS) method to randomly select participants to take part in the survey (See ref. ^11^ for more details). Overall, the sample consisted of 1305 adolescents and young adults and 685 children. Informed consent was taken prior to administering the questionnaire from the adult participants themselves and from a parent in case of minor participants.

### Survey instrument for adolescents and young adults

This age group responded to a structured questionnaire. The survey instrument consisted of four main sections. The first section was dedicated to gathering socio-demographic characteristics of respondents, including information such as age, gender, languages spoken, place of birth and residence (before and after arrival in Turkey), religion, and refugee status. The second section focused on assessing various aspects of social cohesion. Social cohesion is a multidimensional concept, and has no unique definition. Many dimensions of social cohesion have been considered in the literature, and many dimensions have been operationalised in multiple ways. Our measures do not comprehensively include all dimensions and operationalisations considered in the literature but represent the six commonly used dimensions identified by Schiefer and van der Noll^16^. Our measure of social cohesion encompassed perceptions related to both the in-group and out-group. Building on the work by Kuhnt *et al*.^9^ and WFP^17^, we collected data on three key dimensions of social cohesion – *belongingness*, *trust* and *relational capacity*.

For the first dimension, respondents were queried about their sense of belongingness concerning ten different groups. These encompassed family, people of the same age, religion, gender, interests, as well as those residing in the same neighbourhood, in Turkey, those sharing the same country of origin and language. For the second dimension, respondents reported their level of trust towards seven groups, including their family, friends, strangers, neighbours, Syrian nationals, Turkish nationals and people of a third (non-Syrian, non-Turkish) nationality. The third dimension can be further divided into two sub-categories. The first sub-category assessed willingness to form friendships, with respondents indicating their *willingness to befriend* Turkish nationals, Syrian nationals and a third (non-Syrian non-Turkish) nationality. The second sub-category focused on interaction and sharing communal spaces. Within this sub-category, respondents were asked about their willingness to collaborate with others, engage in learning activities together, provide assistance to others, and work jointly with others to address societal challenges. Importantly, this subcategory captured general perceptions regarding fellow members of society without specifying nationality. For each group within each dimension, respondents chose their response from a 4-point hedonic scale – strongly agree, agree, disagree and strongly disagree. Furthermore, this section included inquiries related to which group elicited the strongest sense of belonging, perceptions of safety, and prospects in Turkey.

The third section delved into the educational background of the respondents, as well as their perceptions of their classmates and teachers. Finally, the fourth section was dedicated to gathering data regarding the employment status of the respondents, including whether they had received informal or vocational training.

### Survey instrument for children

The survey instrument for children consisted of two primary sections. The first gathered basic information about the respondents, including details such as age, gender, languages spoken, place of residence before arriving in Turkey and respondent’s openness to making friends with children who speak different language than them. The second section focused on eliciting perceptions. Since perception-based questions (as discussed above) could be too abstract and cognitively challenging for this age-group, behavioural games were used to elicit their perceptions with respect to altruism (dictator game) and trust (trust game). Each child played only one game, with both games having two rounds – one with Turkish partner and one with Syrian partner. To do this, the enumerators paired participants to a randomly selected Turkish child and a randomly selected Syrian child. The identity of the paired child was kept anonymous from the respondent, except for their nationality which was required for the game. In both games, respondents allocated token between themselves and their partner. At the end of the activity, participants exchanged the tokens they won for stickers.

In the dictator game, each respondent received an endowment of four tokens at the beginning of each round, and they had to allocate these tokens between themselves and their partner. After making the choice, the tokens were placed in an envelope.

We employed a simplified version of the trust game, primarily due to time and organizational constraints. Unlike the previous game, there were no initial endowments provided to the participants. Instead, respondents were presented with two choices in both rounds, from which they had to select one. The first option allowed them to retain two tokens for themselves while allocating none to their partner. The second option entailed sharing four tokens with the partner and leaving the allocation decision entirely to the partner. If a respondent opted for the second choice, they were additionally asked how many tokens they expected the partner would allocate to them.

## Data Records

The data for this study is available on the data depository platform, Göttingen Research Online, of University of Göttingen (10.25625/YORNX5)^18^. The data folder consists of four files, two for each age group. Each file name includes the corresponding demographic group and age to clearly indicate which group they belong to. The questionnaire for each round is provided in English as PDF files, titled “Questionnaire [demographic group] [(age in years)]”. The data for each round is in MS Excel format, titled “raw data [demographic group] [(age in years)]”. Each data file has three tabs: 1) dataset, 2) variable label, and 3) codebook. The data is in its raw format in the first tab ‘dataset’; however, two variables have been changed for anonymity concerns and an additional variable has been created that allows easier usage of data. The data set recorded the name of the volunteers and the activity they organised. To maintain anonymity the names in both these variables have been converted to numerical codes using Stata 17.0 (using egen command and manual corrections to account for spelling differences in name of activity). Each volunteer organised an activity for the participants of the BILSY program, which was called an event. Since activity names and volunteer names are not unique, both of these variables cannot identify a unique event independently. However, the joint information provided by the volunteer and the activity ID allows us to identify each event uniquely. Therefore, using Stata 17.0 (egen, group command) we create a variable named “event” which provides a unique ID for each event in a city. This variable has been clearly indicated in the tab ‘variable label’. Data users can either use the variable “event”, or “volunteer_ID” and “activity_ID” together to identify each event. The first column in tab ‘variable label’ provides the name of the variable and the second column presents the description of the variable. In the third column it is indicated if the variable was collected during the survey or created post survey. In the tab ‘codebook’ the response categories and their detailed description are provided.

## Technical Validation

The survey tools underwent a pilot phase between August and September 2018 to ensure their effectiveness and reliability. During this phase, several key aspects of the questionnaires were carefully examined and refined. This included translation accuracy and clarity of questions so that any potential sources of confusion or ambiguity in the wording were identified and addressed to ensure consistent interpretation by respondents. This phase was also central to improve the design of behavioural games and make sure children could easily understand the questions and the rules of the games. The sensitivity of the survey topic, particularly when engaging with marginalized groups, required special attention. Therefore, enumerators underwent comprehensive training to approach respondents with cultural competence and respect for their beliefs and traditions. This training equipped enumerators with the skills needed to conduct the survey in a culturally sensitive manner. The data collection was monitored daily from remote to detect any anomality in the data. Furthermore, for the entire duration of the data collection, field supervisors monitored the data collection in person.

Data was collected on mobile tablets with pre-programmed plausibility checks for logical patterns and consistency. For example, if a child participated in dictator game, questions related to trust game would be automatically skipped on the tablet. These skip patterns used in data collection have been provided in the questionnaire file. Continuing from the example above, in the child questionnaire for question q2_1 if dictator game is selected, then only questions q2_2 and q2_3 are asked. Questions q2_4 – q2_7 are skipped as shown by the skip instruction in the questionnaire. If trust game is selected, the questionnaire asks to skip to q2_4, and ignore q2_2 and q2_3. These skip patterns and instructions are included in the questionnaires for both age-groups.

## Data Availability

No custom code was used in generating the dataset.

## References

[1] Ullah, A. K. M. Conflicts and displacements in Syria: Exploring life trajectories of separated refugee minors. *Asian Journal of Middle Eastern and Islamic Studies* **12**(2), 207–224, 10.1080/25765949.2018.1485922 (2018). Ahsan.

[2] UNHCR. Global Trends Report. Available online at https://www.unhcr.org/globaltrends.html (Accessed on 24^th^ August 2022) (2022).

[3] DGMM. Directorate General of Migration Management: Temporary protection, Republic of Turkey Ministry of Interior, https://en.goc.gov.tr/temporary-protection27 (Accessed on 20^th^ October 2021) (2020).

[4] İçduygu, A. Syrian refugees in Turkey: The long road ahead. Washington, DC: Migration Policy Institute https://www.migrationpolicy.org/research/syrian-refugees-turkey-long-road-ahead (2015).

[5] Korkut, U. Pragmatism, moral responsibility or policy change: the Syrian refugee crisis and selective humanitarianism in the Turkish refugee regime. *Comparative Migration Studies* **4**(1), 1–20, 10.1186/s40878-015-0020-9 (2016).

[6] Erdoğan, M. M. Syrians barometer 2017: A Framework for Achieving Social Cohesion with Syrians in Turkey https://mmuraterdogan.files.wordpress.com/2017/12/sb-2017-executive-summary-06122017.pdf (cited with permission) (2017).

[7] Erdoğan, M. M. Syrians Barometer 2019: A Framework for achieving social cohesion with Syrians in Turkey. *UNHCR* https://39930e27-562f-45ee-8eca-5a5dea2fbdb6.filesusr.com/ugd/c99bb3_73fb751e51624c37b3117b98521e3205.pdf (2020).

[8] International Crisis Group. Turkey’s Syrian Refugees: Defusing Metropolitan Tensions. No. 248. https://www.crisisgroup.org/europe-central-asia/western-europemediterranean/turkey/248-turkeys-syrian-refugees-defusing-metropolitan-tensions (International Crisis Group 2018).

[9] Kuhnt, J., Rischke, R., David, A. & Lechtenfeld, T. Social cohesion in times of forced displacement-the case of young people in Jordan. *Zeitschrift für Flucht- und Refugeesforschung* **3**(2), 320–342, 10.5771/2509-9485-2019-2-320 (2019).

[10] Barron, K., Harmgart, H., Huck, S., Schneider, S. O. & Sutter, M. Discrimination, narratives and family history: An experiment with Jordanian host and Syrian refugee children. *The Review of Economics and Statistics* 1–34. 10.1162/rest_a_01090 (2021).

[11] Mittal, N., Parigi, M. & Vollmer, S. Social cohesion among Syrian and Turkish children, adolescents, and young adults in Turkey. *International Migration* **63**(3), e13346, 10.1111/imig.13346 (2024). 10.1038/s41597-025-05744-9 PMC12350787 40804348

[12] UNHCR. UNHCR Turkey – Fact Sheet July 2019. Available online at https://reliefweb.int/report/turkey/unhcr-turkey-fact-sheet-july-2019 (Accessed on 24^th^ August 2022) (2019).

[13] Alan, S., Baysan, C., Gumren, M. & Kubilay, E. Building social cohesion in ethnically mixed schools: An intervention on perspective taking. *The Quarterly Journal of Economics* **136**(4), 2147–2194, 10.1093/qje/qjab009 (2021).

[14] Alan, S., Duysak, E., Kubilay, E. & Mumcu, I. Social Exclusion and Ethnic Segregation in Schools: The Role of Teacher’s Ethnic Prejudice. *The Review of Economics and Statistics* **105**(5), 1039–1054, 10.1162/rest_a_01111 (2023).

[15] Boucher, V., Tumen, S., Vlassopoulos, M., Wahba, J. & Zenou, Y. Ethnic Mixing in Early Childhood: Evidence from a Randomized Field Experiment and a Structural Model. IZA DP No. 14260 (2021), https://www.econstor.eu/handle/10419/236291 Institute of Labour Economics: Bonn (2021).

[16] Schiefer, D. & Van der Noll, J. The essentials of social cohesion: A literature review. *Social Indicators Research* **132**, 579–603, 10.1007/s11205-016-1314-5 (2017).

[17] World Food Program. Social Cohesion in Turkey: Refugee and host community online survey, rounds 1-2-3. https://docs.wfp.org/api/documents/WFP-0000073545/download/?_ga=2.3715768.945440768.1636642203-1392758971.1636642203&_gac=1.250331764.1636642223.CjwKCAiAm7OMBhAQEiwArvGi3BfFOuhDss6z3kf9fCTvBLBYCIpVBmnArZnCfqvVEgfVxHgwxUPhlRoChTIQAvD_BwE Ankara, Turkey: United Nations World Food Programme Turkey Country Office (2018).

[18] Parigi, M., Mittal, N. & Vollmer, S. Social cohesion among Syrian and Turkish children, adolescents, and young adults in Turkey: Data from Turkey. *GRO.data.*10.25625/YORNX5 (2025). 10.1038/s41597-025-05744-9 PMC12350787 40804348

